# Supplementary material for: Occurrence, Virulence and Antimicrobial Resistance-Associated Markers in Campylobacter Species Isolated from Retail Fresh Milk and Water Samples in Two District Municipalities in the Eastern Cape Province, South Africa
Source: Antibiotics (Basel). 2020 Jul 21;9(7):426. doi: 10.3390/antibiotics9070426 (PMC7400711; doi:10.3390/antibiotics9070426)
Supplement: Supplementary file 1 [file antibiotics-09-00426-s001.pdf]

Supplementalry Table

Table S1. Primers used for confirmation, characterization and amplification of virulence genes.

Targeted Strains	Primer Sets	Targeted Genes	Base Pairs	PCR Condition	Cycles	Ref.
Genus *Campylobacter*	F: GGTGTAGGATGAGACTATATA<br>R: TTCCATCTGCCTCTCCC	Parts of 16S rRNA	439 bp	95°, 94°, 58°, 72°, 72°<br>5′, 30″, 1′, 1′, 2′	33	[35]
C. fetus	F: GGTAGCCGCAGCTGCTAAGAT<br>R: AGCCAGTAACGCATATTATAGTAG	*cstA*	359 bp	95°, 94°, 58°, 72°, 72°<br>15′, 30″, 1′30″, 1′, 7′	25	[36]
C. coli	F: GCTTCGCATAGCTAACAT<br>R: GGTATGATTTCTACAAAGCGAG	*asK*	502 bp	95°, 95°, 50°, 72°, 72°<br>15′, 30″, 1′30″, 1′, 7′	25	[36]
C. lari	F: TAGAGAGATAGCAAAAGAGA<br>R: TACACATAATAATCCCACCC	*glyA*	251 bp	95°, 95°, 46°, 72°, 72°<br>6′, 30″, 30″, 30″ 7′	30	[36]
C. jejuni	F: CAAATAAAGTTAGAGGTAGAATGT<br>R: CCATAAGCACTAGCTAGCTGAT	*cj0414*	161 bp	95°, 95°, 50°, 72°, 72°<br>15′, 30″, 1′30″, 1′, 7′	25	[36]
Flagellin adherence & colonization	F: GGATTTCGTATTAACACAAATGGTGC<br>R: CTGTAGTAATCTTAAAACATTTTG	*flaA*	1723 bp	95°, 94°, 50°, 72°, 72°<br>4′ , 1′ , 1′, 1′, 5′	35	[3]
C. adherence gene	F: TTGAAGGTAATTTAGATATG<br>R: CTAATACCTAAAGTTGAAAC	*cadF*	400 bp	95°, 94°, 42°, 72°, 72°<br>4′ , 1′ , 1′, 1′, 5′	35	[3]
Invasion associated marker	F: GCGCAAAATATTATCACCC<br>R: TTCACGACTACTATGCGG	*iam*	519 bp	95°, 94°, 47°, 72°, 72°<br>4′ , 1′ , 1′, 1′, 5′	35	[37]
C. invasion protein subunit B	F: TTTCCAAATTTAGATGATGC<br>R: GTTCTTTAAATTTTTCATAATGC	*ciaB*	1165 bp	95°, 94°, 43°, 72°, 72°<br>4′ , 1′ , 1′, 1′, 5′	35	[38]
Flagellia synthesis & modification	F: GAGCGTTTAGAATGGGTGTG<br>R: GCCAGGAATTGATGGCATAG	*flgR*	390 bp	95°, 94°, 50°, 72°, 72°<br>4′ , 1′ , 1′, 1′, 5′	35	[39]
Cytolethal distending toxin subunit B	F: GTTGGCACTTGGAATTTGCAAGGC<br>R: GTTAAAATCCCCTGCTATCAACCA	*cdtB*	495 bp	95°, 94°, 55°, 72°, 72°<br>4′ , 1′ , 1′, 1′, 5′	35	[3]

Table S2. Primers sequences used for screening for antimicrobial resistance genes.

Antibiotics	Primer	Primer Sequence (5′–3′)	Base Pair	PCR Conditions	Cycle	Ref.
Tetracycline	*tetA*	F: GCTACATCCTGCTTGCCTTC<br>R: CATAGATCGCCGTGAAGAGG	201 bp	94°, 94°, 55°, 72°, 72°<br>5′ , 1′ , 1′, 1.5′, 5′	35	[42]
	tetB	F: TTGGTTAGGGGCAAGTTTTG<br>R: GTAATGGGCCAATAACACCG	359 bp	94°, 94°, 55°, 72°, 72°<br>5′ , 1′ , 1′, 1.5′, 5′	35	[42]
	tetC	F: CTTGAGAGCCTTCAACCCAG<br>R: ATGGTCGTCATCTACCTGCC	418 bp	94°, 94°, 55°, 72°, 72°<br>5′ , 1′ , 1′, 1.5′, 5′	35	[42]

	tetD	F: AAACCATTACGGCATTCTGC<br>R: GACCGGATACACCATCCATC	300 bp	94°, 94°, 55°, 72°, 72°<br>5′ , 1′ , 1′, 1.5′, 5′	35	[42]
	tetK	F: GTAGCGACAATAGGTAATAGT<br>R: GTAGTGACAATAAACCTCCTA	460 bp	94°, 94°, 55°, 72°, 72°<br>5′ , 1′ , 1′, 1.5′, 5′	35	[43]
	tetM	F: AGT GGA GCG ATT ACA GAA<br>R: CAT ATG TCC TGG CGT GTC TA	158 bp	94°, 94°, 55°, 72°, 72°<br>5′ , 1′ , 1′, 1.5′, 5′	35	[43]
Ciprofloxacin	*gyrA*	F: CGCGTACTATACGCCATGAACCTA<br>R: ACCGTTGATCACTTCGGTCAGG	441 bp	95°, 94°, 55°, 72°, 72°<br>3′ , 1′ , 1′, 1.5′, 5′	35	[44]
Erythromycin	*ermB*	F: CGAGTGAAAAAGTACTCAACC<br>R: GGCGTGTTTCATTGCTTGATG	320 bp	94°, 94°, 55°, 72°, 72°<br>3′ , 1′ , 1′, 1′, 10′	35	[45]
Chloramphenicol	*catI*	F: AGTTGCTCAATGTACCTATAACC<br>R: TTGTAATTCATTAAGCATTCTGCC	320 bp	94°, 94°, 50°, 72°, 72°<br>5′ , 30″ , 30″, 1.5′, 5′	30	[46]
	catII	F: ACACTTTGCCCTTTATCGTC<br>R: TGAAAGCCATCACATACTGC	543 bp	94°, 94°, 50°, 72°, 72°<br>5′ , 30″ , 30″, 1.5′, 5′	30	[46]
Gentamycin	*aac(3)-IIa (aacC2)*[a]	F: CGGAAGGCAATAACGGAG<br>R: TCGAACAGGTAGCACTGAG	740 bp	94°, 94°, 50°, 72°, 72°<br>5′ , 30″ , 30″, 1.5′, 5′	30	[46]
Ampicillin	*ampC*	F: TTCTATCAAMACTGGCARCC<br>R: CCYTTTTATGTACCCAYGA	550 bp	94°, 94°, 45°, 72°, 72°<br>4′ , 45″ , 45″, 45″, 7′	30	[47]
	GES-1-9 GES-11	F: AGTCGGCTAGACCGGAAAG<br>R: TTTGTCCGTGCTCAGGAT	399 bp	94°, 94°, 55°, 72°, 72°<br>10′ , 40″, 40″, 1′, 7′	30	[48]
	OXA-48-like	F: GCTTGATCGCCCTCGATT<br>R: GATTTGCTCCGTGGCCGAAA	281 bp	94°, 94°, 55°, 72°, 72°<br>10′ , 40″, 40″, 1′, 7′	30	[48]
Imipenem	IMP	F: TTGACACTCCATTTACDG<br>R: GATYGAGAATTAAGCCACYCT	139 bp	94°, 94°, 57°, 72°, 72°<br>10′ , 40″, 40″, 1′, 7′	30	[48]
	VIM	F: GATGGTGTTTGGTCGCATA<br>R: CGAATGCGCAGCACCAG	390 bp	94°, 94°, 57°, 72°, 72°<br>10′ , 40″, 40″, 1′, 7′	30	[48]
	KPC	F: CATTCAAGGGCTTTCTTGCTGC<br>R: ACGACGGCATAGTCATTTGC	538 bp	94°, 94°, 57°, 72°, 72°<br>10′ , 40″, 40″, 1′, 7′	30	[48]
